# Quantum Computational, Spectroscopic (FT-IR, FT-Raman, NMR, and UV–Vis) Hirshfeld Surface and Molecular Docking-Dynamics Studies on 5-Hydroxymethyluracil (Monomer and Trimer)

**DOI:** 10.3390/molecules28052116

**Published:** 2023-02-24

**Authors:** Mohit Kumar, Gautam Jaiswar, Mohd. Afzal, Mohd. Muddassir, Abdullah Alarifi, Aysha Fatima, Nazia Siddiqui, Rashid Ayub, Naaser A. Y. Abduh, Waseem Sharaf Saeed, Saleem Javed

**Affiliations:** 1 Department of Chemistry, Institute of Basic Science, Khandari, Dr. Bhimrao Ambedkar University, Agra 282002, Uttar Pradesh, India; 2 Department of Chemistry, College of Science, King Saud University, Riyadh 11451, Saudi Arabia; 3 S.O.S in Chemistry, Jiwaji University, Gwalior 474011, Madhya Pradesh, India; 4 Department of Chemistry, Dayal Bagh Educational Institute, Agra 282005, Uttar Pradesh, India; 5 Department of Science Technology Unit, King Saud University, Riyadh 11451, Saudi Arabia; 6 Restorative Dental Sciences Department, College of Dentistry, King Saud University, Riyadh 11545, Saudi Arabia; 7 Department of Chemistry, University of Allahabad, Prayagraj 211002, Uttar Pradesh, India

**Keywords:** uracil, DFT calculation, molecular docking, molecular dynamic simulation, cancers, thermodynamic properties

## Abstract

For many decades, uracil has been an antineoplastic agent used in combination with tegafur to treat various human cancers, including breast, prostate, and liver cancer. Therefore, it is necessary to explore the molecular features of uracil and its derivatives. Herein, the molecule’s 5-hydroxymethyluracil has been thoroughly characterized by NMR, UV–Vis, and FT-IR spectroscopy by means of experimental and theoretical analysis. Density functional theory (DFT) using the B3LYP method at 6-311++G(d,p) was computed to achieve the optimized geometric parameters of the molecule in the ground state. For further investigation and computation of the NLO, NBO, NHO analysis, and FMO, the improved geometrical parameters were utilized. The potential energy distribution was used to allocate the vibrational frequencies using the VEDA 4 program. The NBO study determined the relationship between the donor and acceptor. The molecule’s charge distribution and reactive regions were highlighted using the MEP and Fukui functions. Maps of the hole and electron density distribution in the excited state were generated using the TD-DFT method and PCM solvent model in order to reveal electronic characteristics. The energies and diagrams for the lowest unoccupied molecular orbital (LUMO) and the highest occupied molecular orbital (HOMO) were also provided. The HOMO–LUMO band gap estimated the charge transport within the molecule. When examining the intermolecular interactions in 5-HMU, Hirshfeld surface analysis was used, and fingerprint plots were also produced. The molecular docking investigation involved docking 5-HMU with six different protein receptors. Molecular dynamic simulation has given a better idea of the binding of the ligand with protein.

## 1. Introduction

One of the important derivatives of methyl uracil is 5-hydroxymethyluracil (5-HMU). The heterocyclic category includes uracil and its derivatives. Since some of them are the primary building blocks of DNA and RNA such as pyrimidine, cytosine, and uracil, and their derivatives are of prime value. Researchers in the fields of spectroscopy, medicine, and industry have all paid close attention to pyridine-derived biomolecules because of their biological activities [1,2,3,4,5,6].

Reactive oxygen species are created by cells, which damage biomolecules and are crucial to the development of cancers and aging. The creation of 8-oxo-7,8-dihydroguanine, 5-HMU, 5-hydroxyuracil (5-HU), and other major forms occurs as a result of oxidative DNA damage when nucleotides are hydroxylated. As indicators of oxidative DNA damage, hydroxylated DNA adducts have been studied [7]. Although the biological effects of 5-HMU in humans are not completely known, certain glycosylase activities that remove 5-HMU from DNA have been found [8]. During the T-transition mutations, the conversion of 5-methylcytosine to 5-HMU is frequently detected in cancers. High level of 5-HMU formation in tissue DNA has been linked to human disorders and, hence, is regarded as a biomarker for breast cancer and oxidative DNA damage [9,10].

**Scheme 1:**
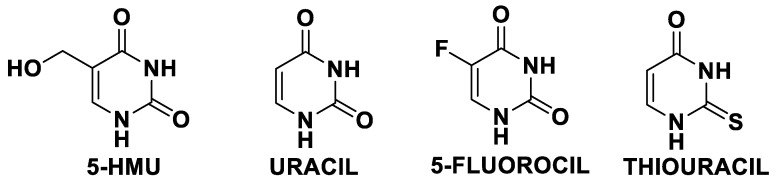
Uracil and its derivatives.

The pharmacological properties of uracil and its derivatives (Scheme 1) have served as the motivation for recent spectroscopic studies [11]. The formation of 5-HMU takes place by the attack of carbonyl oxygen on the DNA’s methyl group. Hydroxy radicals may bring on different modifications to DNA bases. A big aspect of the development of matters is to explore the hydrogen bonding interaction between the 5-HMU and base pairs of DNA [11]. The excited state processes (internal conversion and intersystem crossover) in the fields of intramolecular charge transfer have significantly contributed to analyzing their kinetics of hydrogen bonding resulting from vibrations of the hydrogen donor and acceptor groups [12,13,14].

As far as researchers know, there has not been any literature-reported computational vibrational research on the 5-HMU’s structure. In this study, DFT/ B3LYP theoretical methods with the “6-311++G(d,p) basis set” were employed to gain a comprehensive description of the molecular structure and vibrational pattern of 5-HMU along with experimental analysis (FT-IR, NMR and UV–Vis spectroscopy). Finally, the findings were compared with the theoretical outcomes. The chemicals ampyra, amine dimedone, sulfanilic acid, and sulfathiazole were the focus of similar experiments that we also conducted [15,16,17,18,19].

## 2. Results and Discussion

### 2.1. Optimized Geometry

**Figure 1:**
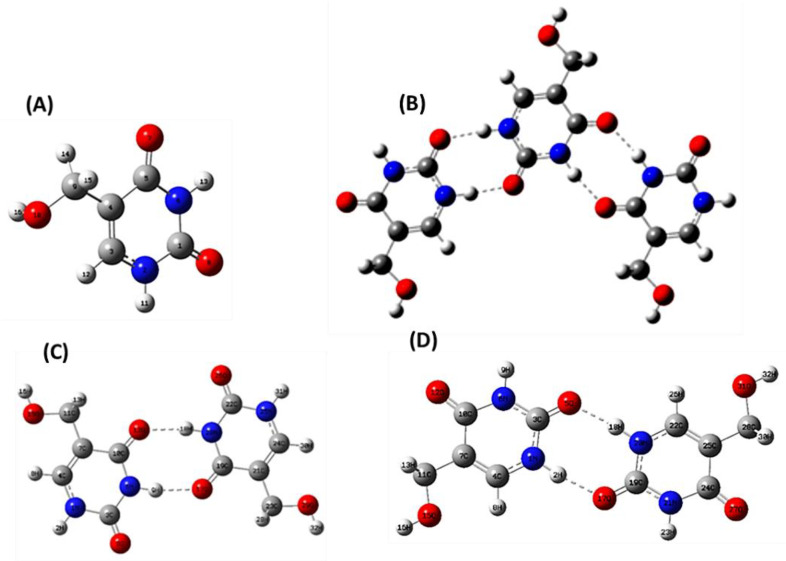
(**A**) The optimized molecular structure of 5-HMU, (**B**) its trimer, (**C**) dimer 1, (**D**) dimer 2.

The optimized geometry of 5-HMU was acquired using the B3LYP methods with the 6-311++G (d, p) basis set, and their comparisons have been made with the experimental analysis (Table 1A,B). The optimized molecular geometry of 5-HMU is shown in Figure 1A and the crystal structure analysis with the diamond program for a trimer (Figure 1B) and two dimers (Figure 1C,D), which was in close resemblance with the structural parameters of the refcode AYEBIP [20,21]. Cagrı et al. have calculated the bond length between C=O, N-C, N-H, O-H, C-C, and C-H as 1.2–1.4, 1.37–1.4, 1.009–1.01, 0.966, 1.3–1.5, and 1.08–1.09 Å, respectively [1]. The obtained values were very close to the experimental parameters which are 1.2–1.4, 1.37–1.4, 1.009–1.01, 0.963, 1.34–1.51, and 1.08–1.09 Å for C=O, N-C, N-H, O-H, C-C, and C-H, respectively. The experimental values for C=O, N-C, N-H, O-H, C-C, and C-H were found to be between 1.23 and 1.4, between 1.36 and 1.38, between 0.87 and 0.89, 0.8, between 1.34 and 1.51, and between 1.08 and 1.1 Å, respectively, which were close to the experimental values of uracil [22]. 

Each 5-HMU molecule is linked to each other via a hydrogen bond to form a trimer unit. In this case, the 2-N-H...O bonds hold within the 5-HMU trimer. The carbonyl and hydroxyl groups drive forming a trimer between monomer complexes in their close proximity. Figure 1 depicts the intermolecular hydrogen bond N-H...O formation based on the optimized structure (bond angles and distances) with the least amount of energy (Table 1B). Because of the inter-molecular interactions, the energy (E) of the trimer was found to be −1588.58 a.u. with N–H…O bond lengths of 1.94 and 1.92 Å (Supplementary Materials, Figure S1). The bond angle of 174.17° and 174.54° in the trimer unit is almost linear. Two independent dimers exhibited by 5-HMU are shown in Figure 1C,D, and the parameters were summarized in Supplementary Materials; Tables S1 and S2.

### 2.2. Vibrational Spectral Analysis

**Figure 2:**
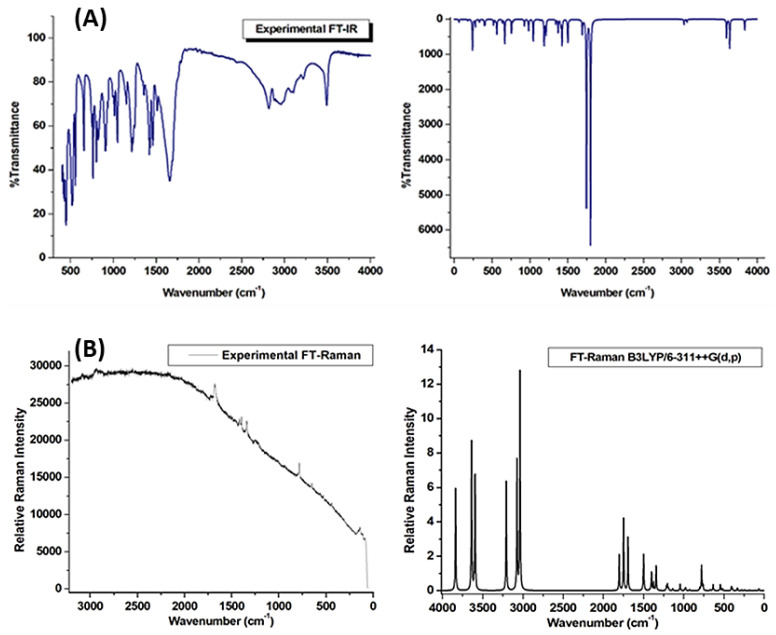
Experimental and computational spectra of (**A**) FT-IR, and (**B**) FT-Raman.

IR spectroscopy may be used to identify chemical groups, and it provides microscopic data on their conformations. IR spectroscopy is a vital method to locate the actual binding regions. Both observed and calculated FT-IR and FT-Raman spectra of 5-HMU were compared in Figure 2A,B. 5-HMU is composed of 14 atoms, resulting in 36 fundamental vibrations modes with C1 point group symmetry, all of which are active in FT-IR and FT-Raman. The estimated frequencies (scaled and unscaled) and PED assignments for 5-HMU on the gaseous phase were assigned by the VEDA software (Table 2).

#### 2.2.1. N-H and C-H Vibrations

By using the B3LYP method and 6-311++G(d,p) method, the bands at 3495 and 3455 cm^−1^ correspond to N-H stretching frequencies, consistent with the experimental value at 3497 cm^−1^ [23]. These pure modes provide 50% and 50% PED, respectively.

The stretching vibration in the FT-Raman spectrum observed at 3068, 2980, and 2917 cm^−1^ for C-H was noticed to be in the range 3083, 2954, and 2919 cm^−1^ in the FT-IR by calculated methods, and their PED assignments contribute to 99%, 94%, and 95% (Table 2).

#### 2.2.2. C-N, H-C-C-C, and C-C Vibrations

The C-N stretching frequency occurs in a composite, which permits the vibrational of various bands [24] In benzamide, the designated C-N stretching band at 1368 cm^−1^ [25]. The stretching vibrations of C-N with a maximum PED assignment of 34% are allocated to the theoretically scaled wavenumbers in FT-IR estimated at 1444, 1438, 1369, 1354, 1166, 1142, 937, 746, and 525 cm^−1^. The FT-Raman band of C-N vibrations was displayed at 1432, 1365, 1175, 1148, 1090, and 514 cm^−1^.

The H-C-C-C torsion vibrations were observed in this investigation, with FT-IR spectra maxima at 1626, 1369, 1290, and 1142 cm^−1^.

The bands in the range of 1400 and 1650 cm^−1^ have been ascribed to vibrations of the C-C heterocyclic ring [26]. In the range of 1300-1000 cm^−1^, stretching vibrations (C-C) are predictable [27]. The C-C stretching vibrations have theoretical frequencies of 1626, 1290, 1166, and 1091 cm^−1^. PED contributes 65%, 15%, 23%, and 11% to various modalities. In the present study, the FT-Raman spectrum shows the stretching vibration of C-C was observed at 1269 and 791 cm^−1^. It demonstrates that theoretical values and experimental data are in good accord.

### 2.3. NMR Analysis

**Figure 3:**
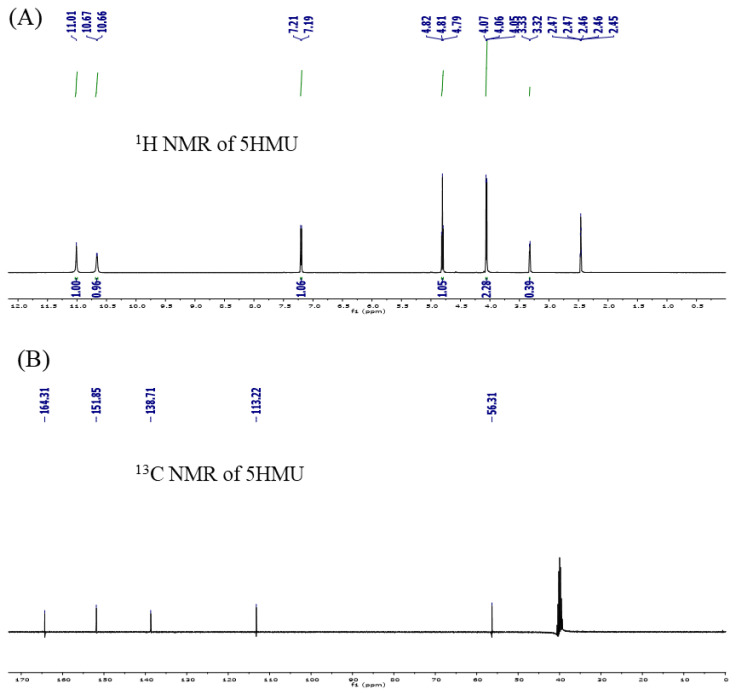
(**A**) Experimental ^1^H and (**B**) ^13^C NMR of 5-HMU in DMSO-*d*_6_ as a solvent.

NMR is a reliable method for obtaining detailed information regarding the structure and chemical environment of organic compounds [28]. Hence, the overall geometry optimization of 5-HMU was analyzed employing the B3LYP method and 6-311++G(d,p) basis set, and with the help of GIAO methodology using the aug-cc-pV5Z def2/J basis set, their chemical shifts value was calculated. The input files have been created with the atomistica online tool [29]. Table 3 shows the actual and estimated chemical shifts for ^1^H and ^13^C NMR. In DMSO solvent, the experimental NMR of 5-HMU were recorded and displayed in Figure 3A,B. In ^1^H NMR, the peaks for hydrogen attached to nitrogen (H11 and H13) were observed at 10.66 and 7.20 ppm and theoretically appeared at 7.06 ppm and 6.75 ppm, respectively. In ^13^C NMR, the carbonyl carbons (C1 and C5) were observed in the area of 164.31 and 151.85 ppm while the peaks for C3 and C4 carbons were observed at 138.71 and 113.22 ppm, and the peak for C9 carbon appeared at 56.31 ppm, and theoretical spectral peaks are found at 165.94, 153.59, 141.71, 120.20, and 62.23 ppm.

### 2.4. Hirshfeld Surface (HS) Analysis

**Figure 4:**
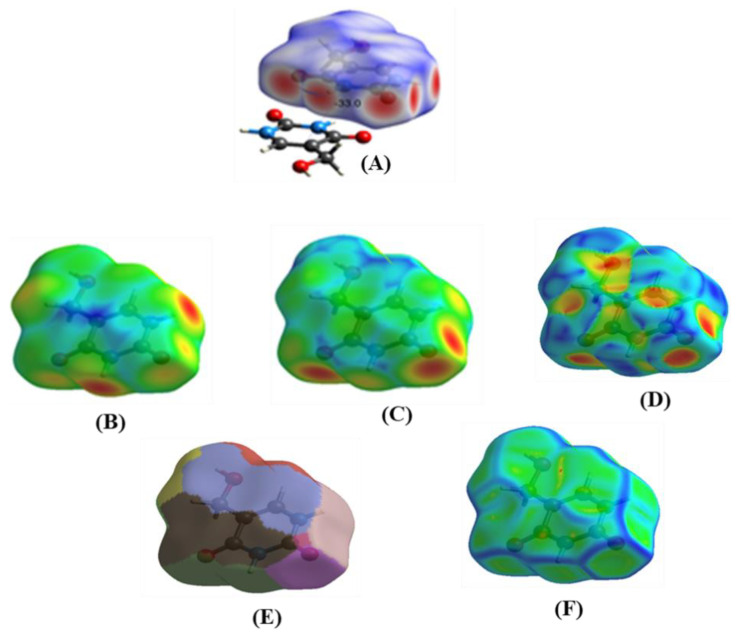
Hirshfeld surface for 5-HMU: mapped with d_norm_ (**A**), d_i_ (**B**), d_e_ (**C**), shape index (**D**) curvedness (**E**), Fragment patch **(F**).

HS analysis was established to divide the crystal’s electron density into covalently electron density pieces. The HS is plotted over d_norm_, a property that has values between −0.6245 (red) and 0.9768 (blue) a.u. [30,31,32,33,34] along with d_i_, d_e_, shape index, curvedness, and fragment patch, respectively (Figure 4). The values of d_i_ ranged from 0.7259 to 2.3342, of d_e_ from 0.7281 to 2.2269, of shape index from −0.9922 to 0.9975, of curvedness from −4.043 to 0.306, and of fragment patch from 0 to 13 (Supplementary Material, Table S3). Intermolecular interactions could be seen using color labeling. Spots in red, white, and blue represent intermolecular interactions that occur at distances that are less than, equal to, and greater than the van der Waal radii, respectively.

The HS and its inside closest nucleus are separated by a distance of d_i_, whereas the closest nucleus outside is separated by a distance of d_e_. The type of weak interactions that the molecules in the crystal packing experience are also quantified using 2-D fingerprint designs [35,36,37]. When calculating individual interatomic interactions, the mutual contact for each interatomic contact is also taken into account. The spikes of 2D FP plot show the interatomic interactions that significantly influence crystal packing (Supplementary Material, Figure S2). The major percentage contributions in the crystal packing belonging to O...H, H...H, and C...O are 44, 27.9, and 10.1%, respectively. The other interatomic connections from C…O, N…H, N…O, C…C, N…C, and O…O are 5.25, 4.6, 4.1, 1.7, 1.3, and 1.2%, respectively, that contribute considerably less to crystal packing.

### 2.5. Molecular Electrostatic Potential Surface (MEPs)

The MEP’s crucial points can be utilized to identify both the structural and electronic characteristics of molecules. It also indicates the shape and size and its positive, negative, and neutral potentials of the molecular system with the help of a color gradient. It is a very useful factor for its electrophilic and nucleophilic properties as well as hydrogen bonding interactions [38]. It also holds importance to verify the proof of the drug’s reaction as an inhibitor and its reaction toward the electrophilic and nucleophilic attacks. This can also be used to predict the relationship between the physicochemical characteristics and molecular structure of the drug [39] such as (a) molecular recognition, drug-receptor interactions; (b) reactive properties of biomolecules; and (c) chemical carcinogenesis, etc. [40,41].

The negative and positive potential sites are preferred for an electrophilic and nucleophilic attack, respectively, corresponding to red and blue colors, respectively. The zero potential sites are encoded in a green color.

**Figure 5:**
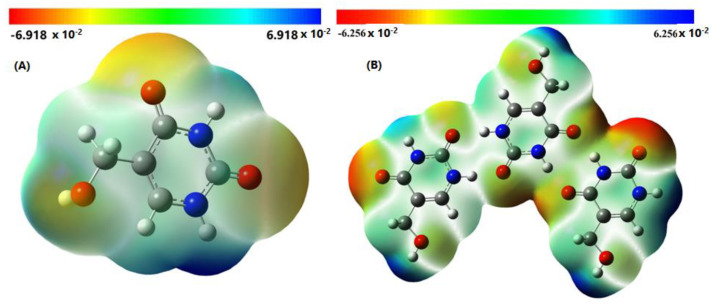
MEP pattern of 5-HMU, (**A**) For Monomer (**B**) For Trimer.

The MEP of 5-HMU and its trimer was calculated using DFT and B3LY/6-311++G(d,p) level of theory and ranged 6.918 × 10^−2^, -6.256 to 6.918 × 10^−2^, 6.256 × 10^−2^ a.u, respectively. The 3D plots of monomer and trimer show that the negatively charged potential. Which is a site of the electrophilic attack, is seen near the oxygen atom while the positive potential (blue color) is found near the carbon and hydrogen atoms (Figure 5A,B). It can be inferred that the reactive sites of the 5-HMU are C=O and nitrogen atoms.

### 2.6. Electron Localization Function

If an electron pair is localized, it means that there is a high possibility of paired electron system with the opposite spin in a certain area of space, leading to the low probability of interaction with outer electrons of this area. A surplus of kinetic energy density generated as a result of Pauli repulsion serves as the foundation for ELF measurement. The baseline of the electron localization function is formed by the electron pairs caused by covalent bonds in molecular space.

**Figure 6:**
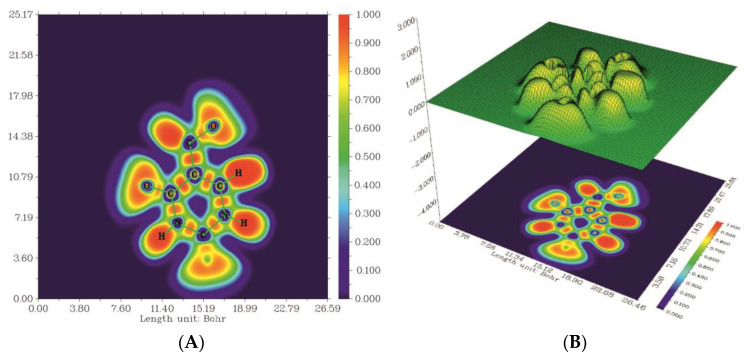
ELF (**A**) Color filled map, (**B**) Shaded surface map with projection of 5-HMU.

ELF can also reveal some important information regarding reactivity, molecular bonding, and chemical structure [42]. Tiny, localized electrons comprise atomic shells, chemical bonds, and lone pair electrons, which governed the strong Pauli repulsion zone. The graph represents high values in a red color (maximum Pauli repulsion) and lower values in a blue color (no Pauli repulsion). The ELF values can be represented graphically in two and three dimensions as a shaded surface map with projection exposing the hydrogen bonding (Figure 6). As we can see, the region around the N-H and C-H bonds has a high ELF value (red) and, hence, a greater chance of finding a spin pair electron while the region around the nucleus of the atoms in the rings shows blue rings.

### 2.7. Non-Linear Optical (NLO) Analysis

DFT methods employing B3LYP/6-311++G(d,p) basis set have been utilized extensively to study the NLO characteristics of organic materials [43,44]. For this purpose, the dipole moment (μD), linear polarizability (α), and first-order hyperpolarizability (β_tot_) were computed; electron correlation, which is dependent on the basis set used, can alter the hyperpolarizability value [45,46,47]. Table 4 lists the computed dipole moment as well as the linear polarizability and first-order hyperpolarizability. The values of α and β from the Gaussian output files are given in atomic units (a.u.), which were then translated into electrostatic units (e.s.u) using the formulas 1 a.u. = 0.184210^−24^ e.s.u for α and 1 a.u. = 8.639610^−33^ e.s.u for β.

The calculated dipole moment for 5-HMU was found to be 3.5115 Debye, and the highest dipole moment is seen in the μ_z_ component. The linear polarizability and first-order hyperpolarizabilities were −0.914 × 10^−23^ and 0.3545 × 10^−30^ e.s.u, respectively, compared to the values of urea which are 0.9771 × 10^−23^ and 0.927 × 10^−30^ e.s.u, respectively.

### 2.8. Natural Bond Orbital (NBO) and Natural Hybrid Orbital (NHO) Analysis

The NBO technique offers knowledge about intra- and inter-molecular interactions between the bonds, as well as a suitable framework for dealing interactions in each filled and virtual orbital area, as well as charge transfer and conjugative interactions in molecular systems [48]. The second-order Fock matrix was employed in the NBO analysis to evaluate the donor–acceptor interactions [49]. An empty non-Lewis orbital replaces the localized NBO of the perfect Lewis structure as a result of the interaction. The stabilization energy E^(2)^ associated with the delocalization *i* → *j* is calculated for each donor (*i*) and acceptor (*j*) as:

$$E^{\left(2\right)}=-n_{\sigma }\frac{{\left(Fij\right)}^{2}}{\varepsilon \sigma^{*}\;-\;\varepsilon \sigma }$$

where (F*_ij_*)^2^, *ε_σ_*, ε_σ_*, and n_σ_ are the Fock matrix element between donor (*i*) and acceptor (*j*), the energies of bonding NBO, anti-bonding NBO, and population in the s (donor) orbital, respectively [50]. The more intense the engagement between electron donors and electron acceptors, or the greater the propensity for electron donors to donate to electron acceptors, the stronger the E^(2)^ value, and the more conjugation throughout the system. A stabilizing donor–acceptor interaction takes place when electron density delocalizes between occupied Lewis type (bond or lone pair) NBO orbitals and formally unoccupied (antibonding and Rydberg) non-Lewis NBO orbitals. NBO analysis was analyzed on a Gaussian 09W [51] package at DFT-B3LYP/6-311++G(d,p) theory level (Supplementary Material, Table S4). The weak departure from precisely localized natural Lewis structure that signifies the primary noncovalent effect is designated by the delocalization of σ-σ* electrons. In 5-HMU, the highest stabilization is shown by the delocalization of π (C3–C4) to π*(C5–O7) of 22.5 kJ/mol. The electron contributing from the lone pair(1) of the nitrogen atom (N2) and N6 to the (C1–O8), which offers a good stabilizing energy of 53.48 and 58 kJ/mol, respectively, are another important interaction connected to the resonance in the molecule. Similarly, the electron contributing from lone pair(1) N6 to π*(C5–O7) gives a moderate stabilization energy of 48.98 kJ/mol.

The NBO, which is composed of its natural atomic hybrids (h_A_ and h_B_), displays sp hybridization, their polarization coefficients (C_A_ and C_B_), and atomic orbital (percentage s-character, p-character) (Supplementary Material, Table S5). The σ(C1-N2) is constituted by the sp^2.16^ of carbon (C1) and sp^1.9^ of nitrogen (N2). The C1 here has 31.61% s character and 68.28% p character, and N2 has 34.43% s character and 65.25% p character. As a result, the NBO C1-N2 is a bond between a C1 sp^2.16^ hybrid and a N2 sp^1.9^ hybrid. The larger polarization coefficient (C_B_ = 0.7915) of the N2 atom compared to the C1 atom (C_A_ = 0.6112) reflects its stronger electronegativity.

σ_CN_ = 0.6112(sp^2.16^) _C1_ + 0.7915(sp^1.9^) _N2_

The p-component of the hybrid described by the polar (ϴ) and azimuthal (Φ) angles is utilized to specify its orientation. The hybrid’s directionality is determined by comparing the central line direction between the two nuclei to the maximum angular amplitude. The bond bends, which is then stated as the angle of divergence between these two directions. Because of the optimization of geometry, the orientation of geometry changes (Supplementary Material, Tables S6 and S7). The carbon NHOs of NBO σ C1-N2 (NBO 1) and σ C1-N6 (NBO 2) are bent away from the line of centers of C-N by 5.3° and 5°, respectively, while the nitrogen NHOs are bent away by 1.2° and 1.4°, respectively.

### 2.9. Population Analysis

Analysis of atomic charge is essential because it determines the atoms NMR chemical shifts and impacts the system’s dipole moment, polarizability of molecules, electronic structure, and molecular reactivity. Using the B3LYP/6-311++G(d,p) method and basis set together with the Mulliken population analysis (MPA) method, the charges on the atoms of the 5-HMU were determined (Supplementary Material, Table S8 and Figure S3). There are five carbon atoms, three oxygen, and two nitrogen atoms as depicted in Figure S3. Almost all carbon atoms are positively charged except C9. Out of the carbon atoms, C1 attached with the hydroxy group has the highest positive charge due to the electron-withdrawing nature of oxygen. C9 was the most negatively charged of all the atoms. The oxygens are almost similar in charge, same with the case of nitrogen atoms. It is usual that all the hydrogen atoms have the same charges with a little difference due to the presence of different groups attached to them.

The Fukui function is a useful tool based on Mulliken population analysis. It gives quantitative information such as the electron densities and determines the most electrophilic and nucleophilic sites on a molecule. The electron density decreases upon addition of electron to some spots providing the negative value of the Fukui function. On the contrary, positive values mean an electron is removed and electron density is increased.

As per Parr and Yang [52], the Fukui function’s value can be used to determine the reactivity. As a result, the condensed Fukui function’s value increases and so does the atomic center’s level of reactivity. When the molecule is attacked by an electrophile or when it loses electrons, the reactivity is denoted by the symbol f-, whereas when it is attacked by a nucleophile or when it gains electrons, the reactivity is denoted by f+. The value of Fukui functions along with the local softness of each atom obtained using Mulliken population analysis has been summarized in Supplementary Material, Table S8. It can be seen that C9 and O8 have the highest values for f^+^ and f^−^, respectively. These sites are likely to undergo nucleophilic and electrophilic attacks, respectively. The reactivity order for electrophilic attack is O8 > C4 > O7 > N2 > O10 > N6 > C5 while the order for nucleophilic attack is C9 > C1 > N2 > C3 > O10 > N6, and the order for radical attack is C9 > C1 > C3. The electrophilic attack is shown to have higher reactivity than the other two.

### 2.10. Frontier Molecular Orbital (FMO) and UV–Vis Spectra

**Figure 7:**
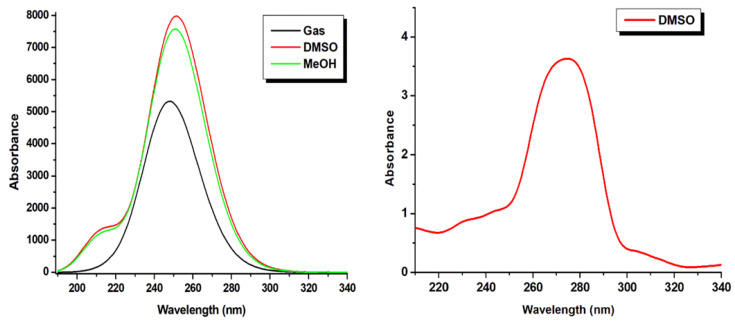
UV–Vis spectra of 5-HMU: Theoretical in the gas phase, DMSO, and Methanol and Experimental in different solvents and experimental in DMSO.

The UV–Vis spectrum of 5-HMU was measured experimentally in DMSO solvent. Using the TD-DFT/B3LYP/6-311++G(d,p) basis set and the PCM model polarizable continuum model, the UV–Vis spectrum was estimated in the DMSO and methanol solvents. The excitation energy, oscillatory strength, and bandgap are summarized in Supplementary Materials; Table S9. The calculated absorption maximum in gas, DMSO, and methanol were 261.67, 252.24, and 251.81 nm, respectively, while the experimental value in DMSO was observed at 275 nm. The calculated and experimental UV–Vis spectra of the titled molecule were given in Figure 7. It is very much clear from the spectra that the molecule shows a (red and hyperchromic) shift in DMSO and MeOH because of secondary interaction with the solvent.

**Figure 8:**
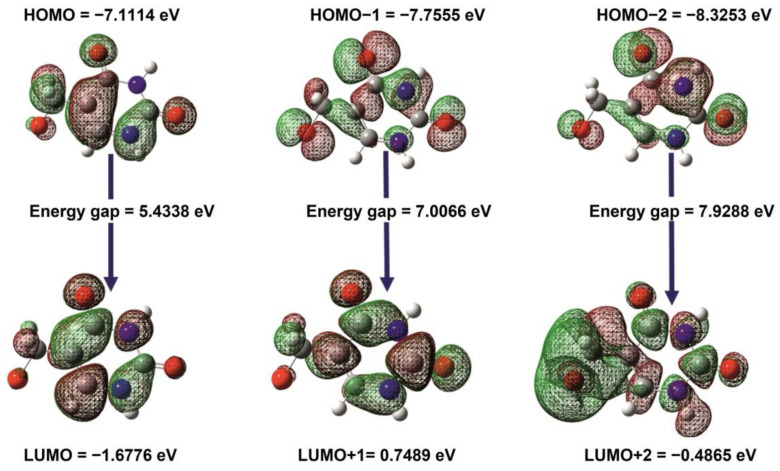
HOMO–LUMO diagrams with band gap energy of 5-HMU.

There are various methods for calculating the excitation energy. The first is the difference between the lowest unoccupied molecular orbital (LUMO) and highest occupied molecular orbital (HOMO) of a neutral system (LUMO). These orbitals regulate the manner in which medications interact with other molecules, such as interactions between pharmaceuticals and their receptors. The HOMO electrons’ propensity to transfer to the LUMO can be accurately determined qualitatively using the frontier molecular orbitals (FMO). The HOMO, LUMO, and FMO gaps, which are crucial aspects of quantum chemistry [53], serve as illustrations of the chemical reactivity and kinetic stability of molecules. While the LUMO orbital mostly functions as an electron acceptor, the HOMO orbital primarily serves as an electron donor. At B3LYP/6-311++G(d,p), the HOMO, LUMO, and orbital energy gaps for the 5-HMU were determined. The highest molecular orbital value that is occupied is −7.12 eV, and the lowest molecular orbital value that is empty is −1.68 eV (Supplementary Materials; Table S10). HOMO-LUMO, HOMO-1-LUMO+1, and HOMO-2-LUMO+2 have energy gaps of 5.4338 eV, 7.0066 eV, and 7.9288 eV, respectively (Figure 8). It can be seen from the plot of HOMO that the positive and negative regions are spread equally all over the molecule same as in the case of LUMO. The charge transfer interaction between the molecules is explained by the energy gap between HOMO and LUMO. The smaller the energy gap (5.44 eV), the easier the charge transfer between the two orbitals due to the electron acceptor’s strong electron-accepting capacity. The bioactivity of the molecule is due to the high charge transfer contact. There are some important tools such as ionization potential (I.E), electron affinity (E.A), electronegativity (χ), chemical hardness (ɳ), and chemical potential to check the order of stability of the molecular system (Supplementary Materials; Table S10). HOMO and LUMO energies are used to compute chemical hardness and chemical potential (μ). A negative chemical potential is defined as electronegativity. Chemical hardness could be a good predictor of chemical stability. Soft molecules are those with a little low energy gap, while hard molecules are those with a big energy gap [54,55].

When the chemical hardness of a substance is raised, it is said to have higher stability. A hard molecule is said to have a wide energy gap when compared to soft molecules [56]. The calculated chemical hardness of 5-HMU is 2.71. The electrophilicity index of can be used to describe a compound’s biological potential, and the calculated electrophilicity index of 5-HMU is 3.55. Furthermore, it has very low softness value of 0.369. It provides information on the toxicity of various contaminants based on their reactivity and site selectivity [57].

### 2.11. Electron Excitation (Electron and Hole Density Distribution) Analysis

Electron and hole density distribution provides the concept of multi-molecular orbital excitation that delivered obvious features of the excited state for the receptors and the corresponding receptor anion. Once an electron is delocalized from A to B as a part of single electron excitation processes (where A and B are real space functions), the excitation of an electron from an occupied to virtual molecular orbital (MO) is taken as a model for excited state. The ground state of MO can be compared to the various regions of photoexcited states shown by the density distribution maps for electrons (ρ^ele^(r)) and holes (ρ^hole^(r)). According to T. Lu and F. Chen, the electron and hole density distribution (EDD and HDD) can be explained by MO wavefunction (Φ) and the configuration coefficient (w), which resembles to the transition of an electron from occupied MO(i) to a virtual MO (1) as shown in equations below [58]:

(1) $$\rho^{\mathrm{ele}}(r)=\sum \;{\left(W\begin{array}{c} l\\ i \end{array}\right)}^{2}\;\Phi_{l}\;(r)\;\Phi_{l}\;(r)+\sum \;\sum \;W\begin{array}{c} l\\ i \end{array}W\begin{array}{c} m\\ i \end{array}\;\Phi_{l}\;(r)\;\Phi_{m}\;(r)$$

*i*→*l*         *i*→*li*→*m* ≠ *l*

(2) $$\rho^{\mathrm{hole}}(r)=\sum \;{\left(W\begin{array}{c} l\\ i \end{array}\right)}^{2}\;\Phi_{i}\;(r)\;\Phi_{i}\;(r)+\sum \;\sum \;W\begin{array}{c} l\\ i \end{array}W\begin{array}{c} l\\ j \end{array}\;\Phi_{i}\;(r)\;\Phi_{j}\;(r)$$

*i→l       i→l j≠i→l*

The electron and hole density distribution maps for 5-HMU were calculated using the aforementioned method. The B3LYP/6-311++G(d,p) level calculations were performed using PCM in DMSO and MeOH and the Multiwfn 3.3.4 software [58].

**Figure 9:**
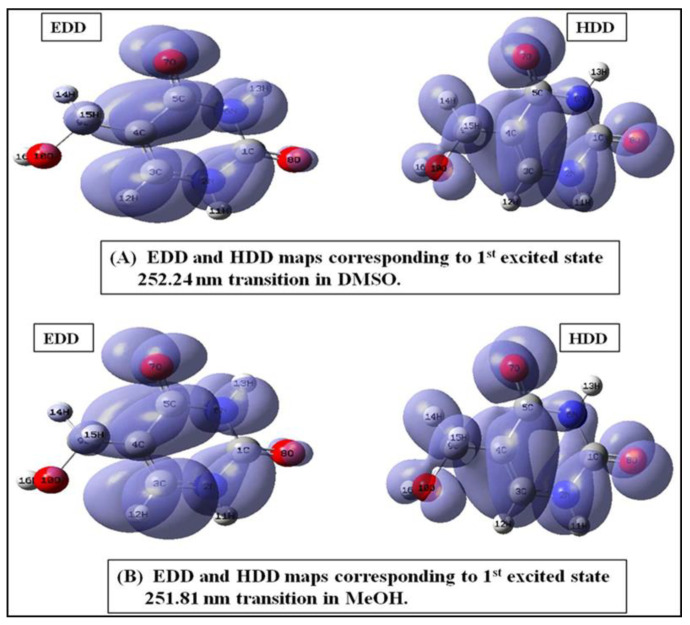
EDD and HDD maps of the excited state of 5-HMU in (**A**) DMSO and (**B**) methanol.

The strong absorption band maxima at 275 nm (4.51 eV) corresponding to HOMO-LUMO were predicted by calculations for the electronic structure of 5-HMU at the TD-DFT- B3LYP/6-311++G(d,p) level with PCM in DMSO. This transition is shown in Figure 9, whereas the absorption at max = 251.81 nm (4.92 eV) for MeOH. The estimated EDD map in Figure 9A depicts a significantly thicker surface on the ring and C=O groups, while the HDD map depicts a denser isosurface on the whole molecule in DMSO. Figure 9B shows EDD and HDD maps created in MeOH; the EDD map exhibits a thicker surface on the entire ring and C=O groups, and the HDD map displays a denser surface on the entire molecule (Supplementary Materials; Table S11).

### 2.12. Thermodynamic Properties

The B3LYP/6-311++G(d,p) technique and basis set by ORCA software were used to calculate the thermodynamic characteristics of 5-HMU. The values of the enthalpy (H^0^), entropy, and Gibbs free energy were computed between 100 K and 700 K. The intensity of molecular oscillations also rises with temperature because of higher translational and rotational energy, which is supported by uniform distribution [59,60]. In light of the fact that thermal stability is crucial both at low and high temperatures, physicochemical effects are observed in the industry to preserve food or medicine storage. As a result, temperature is important in chemical thermodynamics and pharmacodynamics. From the Gibbs free energy, biological thermodynamic applications such as protein stability, membrane, DNA, and enzyme kinetics can be calculated.

The Gibbs free energy (G^0^) shows an inverse relation with temperature, and hence it decreases with an increase in temperature (Supplementary Materials; Figure S4 and Table S12). The correlation between the temperature and the thermodynamic function was determined using quadratic and linear equations. The correlation (R^2^) between the two for entropy, enthalpy, and Gibbs free energy are 1, 0.9999, and 0.9747, respectively. The relationships are plotted in Supplementary Material; Figure S5. The following is the thermodynamic correlation fitting equation:

G = 0.0012x^2^ − 1.3345x + 385.3434 (R^2^ = 0.9747)

S = −0.0001x^2^ + 0.5423x + 214.6058 (R^2^ = 1)

H = 0.0002x^2^ + 0.0298x + 310.3621 (R^2^ = 0.9999)

The thermodynamic properties also influence how non-covalent interactions such as electrostatic interactions, multiple hydrogen bonds, van der Waals interactions, and hydrophobic effects bind to one another [61]. Utilizing the relationship between thermodynamic functions and the second rule of thermodynamics, these features can be used in the thermochemical field to determine the flow of chemical reactions and identify other thermodynamic energy [62].

### 2.13. Molecular Docking

The ligand–protein docking method is used to predict the ligand’s binding sites in proteins using a three-dimensional network. Predicting chemical interactions between the lead compound and biologically relevant targets (proteins, enzymes, etc.) has become critical in the science of drug discovery. Furthermore, by analyzing the docking of the lead molecule with a variety of protein targets, one can gain insight into the underlying molecular mechanisms of selectivity [63].

**Figure 10:**
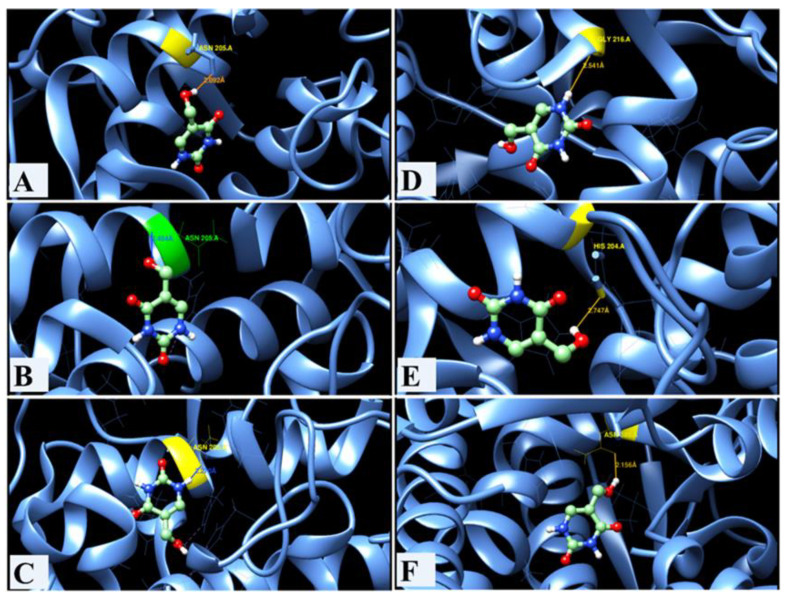
Ligand 5-HMU: embedded in the active sites of (**A**) 4P6T, (**B**) 4P6S, (**C**) 6QXD, (**D**) 6EI4, (**E**) 5OAE, and (**F**) 5M67 proteins.

The two fundamental steps of the docking procedure are the prediction of the ligand structure as well as its orientation within these sites and the evaluation of the binding affinity. The Swiss ADME online source predicted the target protein that needs to dock with the 5-HMU and downloaded the file in RCSB protein database (pdb) format. The relevant target protein ID (docked with a similar ligand as hydantoin) was picked from the Swiss ADME- target prediction site and retrieved from the protein data bank with the aid of a smile string of 5-HMU ligand, which was added to the site of the Swiss target prediction (PDB). The active sites for docking are ligands that have already docked inside proteins. The proteins 4P6T 4P6S, 6QXD, 6EI4, 5OAE, and 5M67 all belonging to the oxidoreductase class except 5M67 and were docked with 5-HMU showing the bond distance of hydrogen bond interaction between 5-HMU and the target (Figure 10A–F). In this docking, all the metal centers of the proteins are avoided in PDB’s. The binding energies have been calculated using AutoDock vina [64,65]. The ligand’s characteristics, including its binding energy and inhibition constant, are collected in Table 5. The lowest binding energy (∆G) and intermolecular energy were found to be −5.0 kcal/mol between 5-HMU and 6QXD in the protein–ligand interaction. The highest binding energy was found for 5M67. The calculated bond distance for the same object is 2.270. The solid lines in the figure depict the hydrogen bonds between the ligand and the targeted protein. Inhibition constants were calculated by *K*_i_ = exp(∆G/RT).

### 2.14. Drug-Likeness

To decide the structural appearances of ligands in order to achieve effective and efficient results, a set of rules known as “drug-likeness” is employed following the Lipinski’s rule of five [66,67]. We looked at the polar surface area (TPSA), molar refractivity, and the quantity of hydrogen bond donors and acceptors (HBD and HBA, respectively) (Supplementary Materials; Table S13), which shows that these metrics have values within acceptable ranges for the medication candidate under evaluation. The HBD and HBA should be less than 5. The title molecule satisfies these requirements. The MLogP, one of the most critical characteristics (showing the hydrophobic/lipophilic nature of the molecule), is equal to −1.22 which is far below Lipinski’s rule of five threshold value of 5. The TPSA value for a compound is 85.95 Å^2^, which is much below the maximum cut-off value of 140 Å^2^ (Supplementary Materials; Figure S6). The molar refractivity was calculated to be 33.81, which falls within the range of pharmaceutical candidates [68].

### 2.15. Molecular Dynamic (MD) Simulation

**Figure 11:**
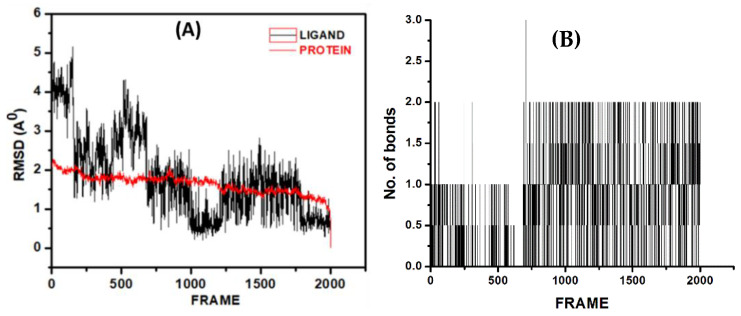
(**A**) Root mean square deviation plot, and **(B**) Hydrogen bond formations.

The efficacy of ligand binding on protein structure was analyzed by using MD modeling. The GROMACS 2022 software was utilized to study MD simulation [69]. The ‘CHARMM36 force field’ was used to generate the protein topology. Each system was solvated using the TIP3P water model before being neutralized with the proper concentrations of Na^+^ and Cl^−^ ions [70]. The docked protein–ligand combination of the molecule with the PDB ID: 6QXD was used to evaluate the stability of the 5-HMU. To confirm the stability of the complex structures, the simulation was run for 20 nanoseconds at room temperature. The system was designed to use as little energy as possible, employing the steepest descent technique. Position restraint together with NVT and NPT ensembles were used after the energy minimization process. Following temperature stabilization, an isothermal isobaric ensemble (NPT) was performed. The trajectory indicates that the docked ligand was stable throughout the simulation in the protein’s binding site where the protein-ligand is relatively stable and exhibits minor conformational changes. The data were examined using the graphical tool origin once all of the MD simulation phases had been completed. The built-in functions of GROMACS 2022 were used to find the different vital parameters, such as root mean square deviation (RMSD), number of hydrogen bonds occurring between protein and the ligand, and interaction energy. The stability of the complex is represented by the RMSD stabilization (Figure 11A). After alignment, further RMSD values were determined for the protein with an inbuilt option in the VMD software; therefore, the protein showed a smaller variation in the RMSD values. Thirteen stable hydrogen bonds are formed between the protein and the ligand, and these bonds define how strong the complex is (Figure 11B). However, in this instance, the total interaction energy is useful. According to the common formula for adding two values, this value is equal to −137.34 ± 11.8 kJ mol^−1^ after propagating the error. According to every outcome of the MD simulation, 5-HMU can bind to the active sites of proteins to create a stable protein complex. The resultant values of MD simulations are more precise and correct in comparison to docking results. We may predict that there is more fluctuation in the RMSD values of ligand arisen due to movement all 13 hydrogen bonds of ligand during the simulation, but not at once. Hydrogen bonds occupancy with their donor–acceptor sides showed in Supplementary Materials; Table S14.

## 3. Experimental Section

### 3.1. Materials and Instrumentations

The spectral study was conducted using the 5-hydroxymethyl uracil (Sigma-Aldrich Delhi, India). The infrared spectrum was measured with FT-IR PerkinElmer (Serial No. 105627) using a KBr disc method (FT-RAMAN at IIT Kanpur, Kanpur, India). NMR spectra were achieved with the help of a Bruker Advance 400/Avll HD-300 (FT-NMR) apparatus. The UV–Vis analysis was carried out on a UV-1280 Spectrophotometer (Shimadzu, Mumbai, India).

### 3.2. Computational Analysis

The supporting information has provided computational details (See the Supplementary Materials).

## 4. Conclusions

The title chemical 5-HMU was examined through the spectroscopic analysis using FTIR, FT-Raman, NMR (^1^H and ^13^C), and UV–Visible in accordance with the theoretical DFT method. The optimized crystal structure of 5-HMU and its trimer that was produced with the least amount of energy were compared to the experimental crystal structure. The development of an intermolecular H-bond with adjacent monomers was found to stabilize the trimer, and its ideal molecular structure was established using the 6-311++G(d,p) basis set and the DFT-B3LYP approach. The complete vibrational assignment was investigated based on potential energy distribution (PED). The nucleophilic and electrophilic sites, as well as the H-bonding interactions of the 5-HMU, were predicted via MEP mapping. The positive potential sites were found on hydrogen and carbon atoms, whereas the negative potential sites were found on oxygen atoms. The electron localization function (ELF) was employed to represent the degree of electron localization. The resonance interaction energy of 5-HMU was 48.98 kJ/mol, as measured by the maximum stabilization of electron leading LP(1) N6—π* C5-O7. The deviation angle, coupled with the polar (θ) and azimuthal (ɸ) angles of the vector from the nucleus, were utilized to determine the orientation of a hybrid. The energy gap between highest occupied and lowest unoccupied was determined to be 5.4338 eV. Three metrics, such as dipole moment, polarizability, and hyperpolarizability, were used to validate a material nonlinear optical characteristic. High chemical hardness (2.72) indicated the compound stability, while low chemical softness (0.37) was designated as non-toxicity, and the electrophilicity index (3.56) indicated 5-HMU biological activity. EDD and HDD maps for the first excited-state were created with DMSO and MeOH to indicate single MO pair excitation. In the temperature range of 100 K to 700 K, the ORCA program was utilized to determine various thermodynamic features. It depicts how the thermodynamic properties change as the temperature increases. The Fukui function describes the intramolecular interaction between atoms in a molecule. The 5-HMU receptor has the lowest binding energy of −5.0 kcal/mol, indicating that further research into the therapeutic potential of the mentioned molecule is recommended. The molecular dynamic simulation gave better idea of binding and showed 11 stable hydrogen bonds.

## Figures and Tables

**Table 1:** (**A**) Optimized geometrical parameters of 5-HMU showing bond length (Å); and bond angles (°). (**B**) Optimized geometrical parameters showing bond length (pm) and bond angle (°) of the trimer.

(**A**)					
Bond length (Å)			Bond angle (˚)		
Parameter	B3LYP/6-311++G(d,p)	Experimental	Parameter	B3LYP/6-311++G(d,p)	Experimental
C1-N2	1.39	1.35	C1-N2-H11	120.93	117.15
C1-N6	1.38	1.36	C3-N2-H11	122.36	120.83
C1-O8	1.21	1.23	N2-C3-C4	116.13	122.70
N2-C3	1.37	1.37	N2-C3-H12	121.50	118.62
N2-H11	1.00	0.87	C4-C3-H12	118.77	118.66
C3-C4	1.34	1.35	C3-C4-C5	122.99	118.01
C3-H12	1.08	0.95	C3-C4-C9	118.21	122.87
C4-C5	1.46	1.45	C5-C4-C9	114.34	119.09
C4-C9	1.51	1.50	C4-C5-6	125.20	115.13
C5-N6	1.40	1.38	C4-C5-O7	120.45	124.63
C5-O7	1.21	1.23	N6-C5-O7	128.00	120.23
N6-H13	1.01	0.89	C1-N6-C5	115.70	126.54
C9-O10	1.42	1.40	C1-N6-H13	116.28	117.05
C9-H14	1.09	0.98	C5-N6-H13	112.81	116.36
C9-H15	1.09	0.99	C4-C9-O10	109.30	108.30
O10-H16	0.96	0.84	C4-C9-H1C4	109.94	110.07
**Bond angle (˚)**			C4-C9-H15	111.89	110.01
N2-C1-N6	112.66	115.48	O10-C9-H14	106.07	110.04
N2-C1-O8	123.22	122.75	O10-C9-H15	106.58	110.02
6-C1-O8	124.11	121.76	H14-C9-H15	108.72	108.38
C1-N2-C3	115.22	122.01	C9-O10-H16	108.72	109.83
R^2^	0.97		R^2^	0.91	
(**B**)					
Bond length			Bond angle		
Parameter	B3LYP/6-311++G(d,p)		Parameter	B3LYP/6-311++G(d,p)	
O17-H2	1.81		N1-H2-O17	174.17	
O5-H18	1.78		N22-H18-O5	174.54	
O12-H20	1.84		N24-H20-O12	170.45	
O19-H9	1.84		N6-H9-O19	170.46	
N22-C21	1.37		C21-O17-H2	125.56	
C21-O17	1.23		C3-O5-H18	126.78	
N22-H18	1.03		C23-O19-H9	132.13	

**Table 2:** Calculated vibrational frequencies (cm^−1^) assignments of 5-HMU.

Mode No	Experimental		Theoretical		I_IR_	I_RAMAN_	Assignments (PED)
	FT-IR	FT-Raman	Unscaled	Scaled			
42	3651		3836	3686	5	48	γOH(100)
41	3491		3637	3495	13	69	γNH(50)
40			3596	3455	9	54	γNH(50)
39	3078	3068	3208	3083	1	51	γCH(99)
38	2960	2980	3074	2954	2	61	γCH(94)
37		2917	3038	2919	3	100	γCH(95)
36		1732	1801	1731	100	17	γOC(48) + βHNC(10)
35	1658	1694	1748	1679	84	34	γOC(44)
34			1692	1626	7	26	γCC(65) + βHCC(11)
33	1458		1503	1444	2	6	γNC(14) + βHNC(16) + βHCH(27)
32	1421	1432	1497	1438	4	16	γNC(10) + βHNC(11) + βHCH(52)
31		1365	1425	1369	1	1	γNC(15) + βHNC(15) + βHCC(11)
30	1357	1357	1409	1354	4	1	γNC(16) + βHNC(46)
29			1399	1345	1	9	γHC(10) + βHNC(11) + τHCCC(42)
28			1374	1320	7	5	βHOC(42) + βHCO(32)
27	1215	1269	1342	1290	3	12	γCC(15) + βHCC(43)
26		1175	1213	1166	7	3	γNC(25) + γCC(23)
25	1151		1201	1154	2	4	βHOC(40) + βHCO(36) + τHCCC(11)
24		1148	1188	1142	12	2	γNC(24) + βHNC(14) +βHCC(19)
23	1047	1090	1136	1091	1	1	γNC(27) + γCC(11) + γOC(13)
22	1013	1035	1044	1003	10	4	γOC(72)
21		945	984	945	5	1	βHOC(10) + τHCCC(43)
20	906		975	937	1	2	γNC(13) + βCNC(23)
19	800	898	926	890	3	1	τHCCC(65)
18	761	791	799	768	1	2	γCC(28) + βCNC(12) + βCCN(10)
17			776	746	1	12	γNC(34)
16			758	728	2	3	τOCCC(30)
15			755	726	5	1	τOCCC(10)
14	659	654	668	641	11	1	τHNCC(66)
13			636	611	2	3	τHNCC(24)
12	559	536	563	541	7	1	τHNCC(69)
11	520	514	546	525	2	3	γNC(10) + βONC(25) + βCNC(29) + βCNC(10)
10	451		520	499	3	1	βCNC(12) + βNCN(32) + βOCC(22)
9			407	391	3	2	βOCN(37) + βCNC(11)
8			398	382	3	1	τCCNC(42)
7			334	321	2	2	βCNC(12) + βOCN(16) + βCCN(14) + βNCN(10) + βOCC(15)
6			282	271	4	1	τHOCC(20)
5			245	235	14	1	τHOCC(75)
4			182	175	1	1	βCCC(63) + βOCC(19)
3			147	141	1	1	τCNCN(77)
2			110	106	1	1	τCCNC(18) + τCNCN(58)
1			66	64	2	1	βCNCN(13) + βOCCC(67)

**Table 3:** Experimental and theoretical ^13^C and ^1^H chemical shift values (ppm) of 5-HMU in DMSO.

Atoms	Experimental	Calculated (B3LYP/6-311++G(d,p))	Calculated (aug-cc-pV5Z def2/J)
C5	164.31	165.946	151.95
C1	151.85	153.5959	128.18
C3	138.71	141.7179	102.59
C4	113.22	120.2056	70.18
C9	56.31	62.2347	61.45
H12	11.01	7.3857	19.54
H13	10.66	7.061	8.46
H11	7.20	6.7589	6.43
H14	4.81	4.6287	6.43
H15	4.06	4.2636	5.03
H16	3.33	0.5378	3.19

**Table 4:** The calculated dipole moment μ(D), polarizability (α;_0_), and first-order hyperpolarizability (β_tot_) components of 5-HMU.

Parameters	B3LYP/6-311++G(d,p) (5-HMU)	B3LYP (Urcail)	Parameters	B3LYP/6-311++G(d,p)
μ_x_	−1.4356		β_xxx_	−41.6733
μ_y_	−3.0482		β_yxx_	21.8308
μ_z_	−0.989		β_xyy_	19.7825
μ(D)	3.5115	1.7615	β_yyy_	−41.3496
α_xx_	−71.1752	93.36	β_zxx_	−13.9748
α_xy_	2.0043		β_xyz_	−7.1467
α_yy_	−58.1887	75.72	β_zyy_	−2.1224
α_xz_	4.4834		β_xzz_	−8.2926
α_yz_	1.4686		β_yzz_	−0.7765
α_zz_	−55.6042	70.11	β_zzz_	−2.9145
α_0_ (e.s.u)	−0.914 × 10^−23^		β_tot_ (e.s.u)	0.3545 × 10^−30^

**Table 5:** Hydrogen bonding and molecular docking score of 5-HMU with protein targets.

Protein (PDB ID)	No of Residues	Bond Distance (Å)	Inhibition Constant (Micromolar)	Binding Energy (kcal/mol)	Reference RMSD (Å)
4P6T	3	2.092	200.89	−5.0	3.620
4P6S	3	2.484	101.67	−5.4	3.690
6QXD	3	2.270	43.40	−5.9	0.000
6EI4	3	2.541	61.01	−5.7	0.000
5OAE	3	2.747	61.01	−5.7	2.256
5M67	3	2.156	61.01	−5.7	4.035

## Data Availability

Data Availability Statements are available in section “MDPI Research Data Policies” at https://www.mdpi.com/ethics (accessed on 30 November 2022).

## Supplementary Materials

The following supporting information can be downloaded at: https://www.mdpi.com/article/10.3390/molecules28052116/s1, Figure S1: Trimer of 5-HMU by a secondary interaction; Figure S2. 2-D fingerprint plot of different contributions for 5-HMU; Figure S3. Graph showing Mulliken atomic charges of 5-HMU; Figure S4. Graph representing the dependence of Entropy, Gibbs free energy, and enthalpy on the temperature of 5-HMU; Figure S5. The plot showing relationships of temperature with Gibbs free energy, and entropy, enthalpy; Graph representing the dependence of Entropy, Gibbs free energy, and enthalpy on the temperature of 5-HMU; Figure S6. Predicted Lipinski’s rule of five showing bioavailability radar plot of 5-HMU; Table S1: Some optimized “Geometrical parameters” Bond Length (pm) and Bond Angle (°) of dimer 1; Table S2. Some optimized “Geometrical parameters” Bond Length (pm) and Bond Angle (°) of dimer 2; Table S3. Surface property information in Hirshfeld for 5-HMU; Table S4. Second-order perturbation theory of the Fock matrix NBO analysis of 5HMU; Table S5. Hybrid, polarization coefficient and atomic orbital contribution in selected natural bond orbitals of 5HMU; Table S6. Natural hybrid orbital directionality and bond bending (deviations from line of nuclear centres) of 5HMU; Table S7. Natural Hybrid Orbital directionality and bond bending (deviations from line of nuclear centres) of 5HMU; Table S8. Mulliken charge distribution, Fukui function, and local softness corresponding to (0,1), (−1,2), and (1,2) charge and multiplicity of 5-HMU; Table S9. Comparison of calculated and experimental electronic properties of 5-HMU; Table S10. Calculated energy values of 5-HMU; Table S11. Calculated centroid coordinates and distances between EDD and HDD centroids for allowed excited state transition of 5-HMU in DMSO and methanol; Table S12. Temperature dependence of thermodynamic properties of 5-HMU at B3LYP/6–311++G(d,p); Table S13. ADME properties of 5-HMU; Table S14. Hydrogen bonds occupancy with their donor-acceptor sides [30,51,58,65,71,72,73,74,75,76,77,78,79,80,81,82].
