# Supplementary material for: Quantum Computational, Spectroscopic (FT-IR, FT-Raman, NMR, and UV–Vis) Hirshfeld Surface and Molecular Docking-Dynamics Studies on 5-Hydroxymethyluracil (Monomer and Trimer)
Source: Molecules. 2023 Feb 24;28(5):2116. doi: 10.3390/molecules28052116 (PMC10004125; doi:10.3390/molecules28052116)
Supplement: Supplementary file 1 [file molecules-28-02116-s001.zip › molecules-2106941-supplementary.pdf]

Supporting Information

Computational Methodology

Density functional theory was used to conduct the quantum computational analysis (DFT). The lower basis set was employed for a finer portrayal of polar bonds. All computations were performed using the B3LYP method and the 6-311++G(d,p) higher-order basis set [1,2]. On a personal computer, the Gaussian 09 W [3] programme package was used for all of the work's computations, and the ORCA 4.0.1 programme was employed for several other useful results [4]. The vibrational wavenumbers and geometrical properties of the molecule were analysed using the B3LYP method with the 6-311++G (d,p) basis set. The potential energy distribution of vibrational frequency was calculated using the vibrational energy distribution analysis (VEDA4) software [5]. The electron localization function (ELF) diagram was generated using the Multiwfn programme software for the topology analysis, according to the atoms in molecule (AIM) theory [6]. The optimised geometry was used to perform the natural bond orbital analysis (NBO). The TDDFT method was used to compute the UV spectrum energies in gas, methanol, and DMSO. In the frame of reference of Koopman's theorem [7], ionisation potential (IP) and electron affinity (EA) are represented by HOMO and LUMO energies:

IP = $-E_{HOMO}$

EA= $-E_{LUMO}$

Electronegativity (χ) = $\frac{IP+EA}{2}$

Chemical potential (μ) = $-\frac{IA+EA}{2}$

Chemical hardness (η) = $\frac{IP-EA}{2}$

Chemical softness (σ) = $\frac{1}{2\eta}$

Electrophilicity index (ω) =$\frac{\mu 2}{2\eta}$

The noncovalent interactions that lead to crystal packing are investigated via Hirshfeld surface analysis. Intermolecular and intramolecular interactions inside the crystal were also studied. According to the hydrogen bond and Hirshfeld surface analyses, the intermolecular interactions are evident in the crystal structure. This approach is based on a 2-D fingerprint plot that summarises the type of contact and a 3-D graph representation in the region of space where the molecules interact. Both qualitative and quantitative analyses [8,9] used the Hirshfeld analysis. Crystal Explorer 17 was used to plot the fingerprint and Hirshfeld surface [10]. Normalized contact (d_{norm}) can be expressed using equation [11]:

$$\{d_{norm} = \frac{d_i - r_i^{vdw}}{r_i^{vdw}} + \frac{d_e - r_e^{vdw}}{r_e^{vdw}}\}$$

The radii of atoms are indicated by ri^{vdw} and re^{vdw} in this diagram (van der Waals).

The interactions in the crystal were evaluated using the distance from the surface to the nearest nucleus (d_i) and the distance from the surface to the atom (d_e) [12]. The di and de express intermolecular interactions via a 2-D fingerprint plot [7]. AutoDock Vina [13] and Chimera [14] were used for molecular docking, and the SwissADME programme [15] was used to assess drug-likeness.

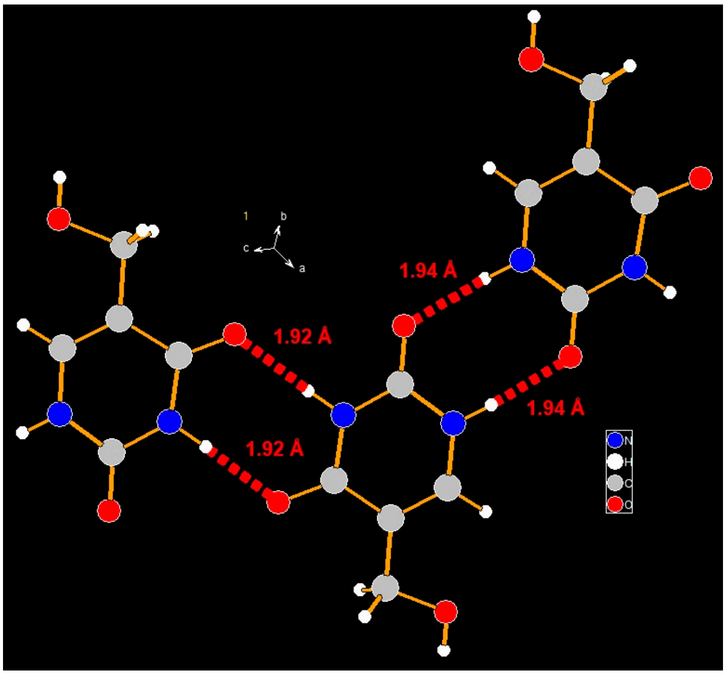

Figure S1: Trimer of 5-HMU by a secondary interaction.

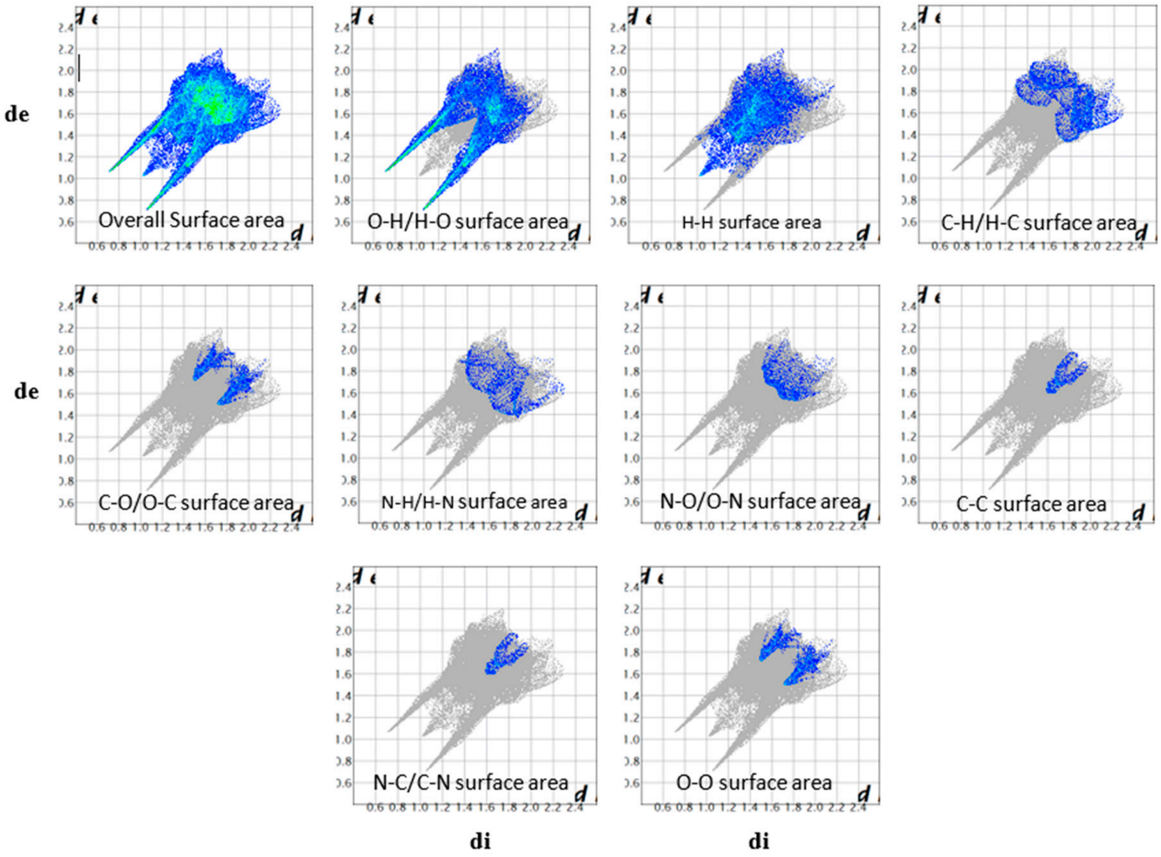

Figure S2. 2-D fingerprint plot of different contributions for 5-HMU.

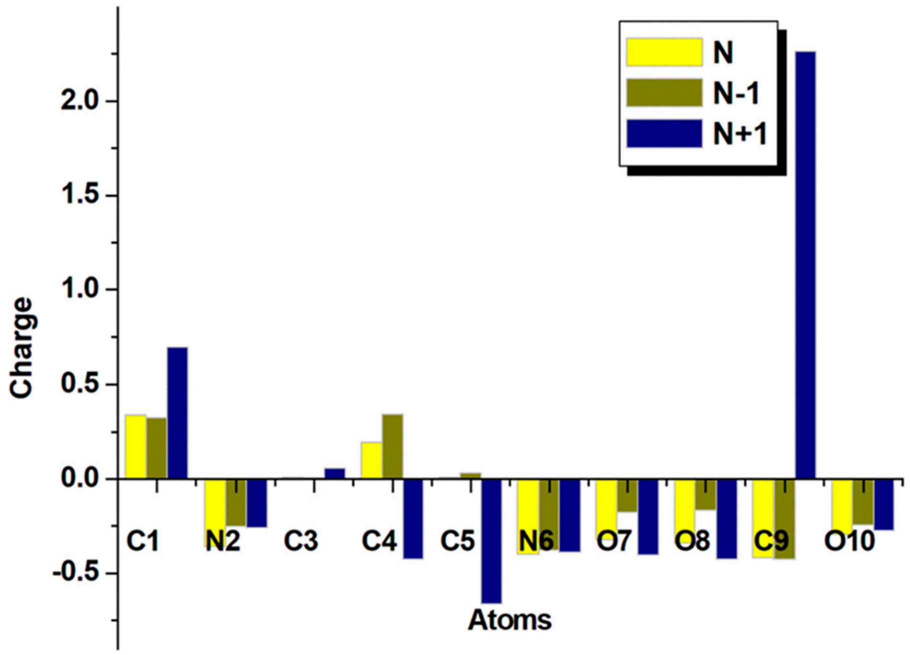

Figure S3. Graph showing Mulliken atomic charges of 5-HMU.

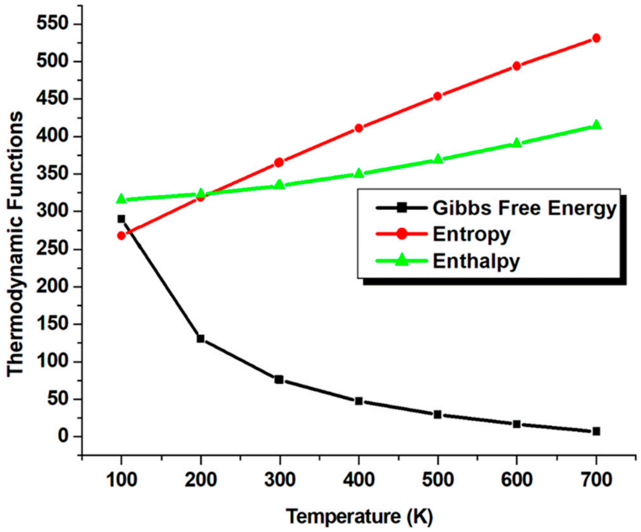

Figure S4. Graph representing the dependence of Entropy, Gibbs free energy, and enthalpy on the temperature of 5-HMU.

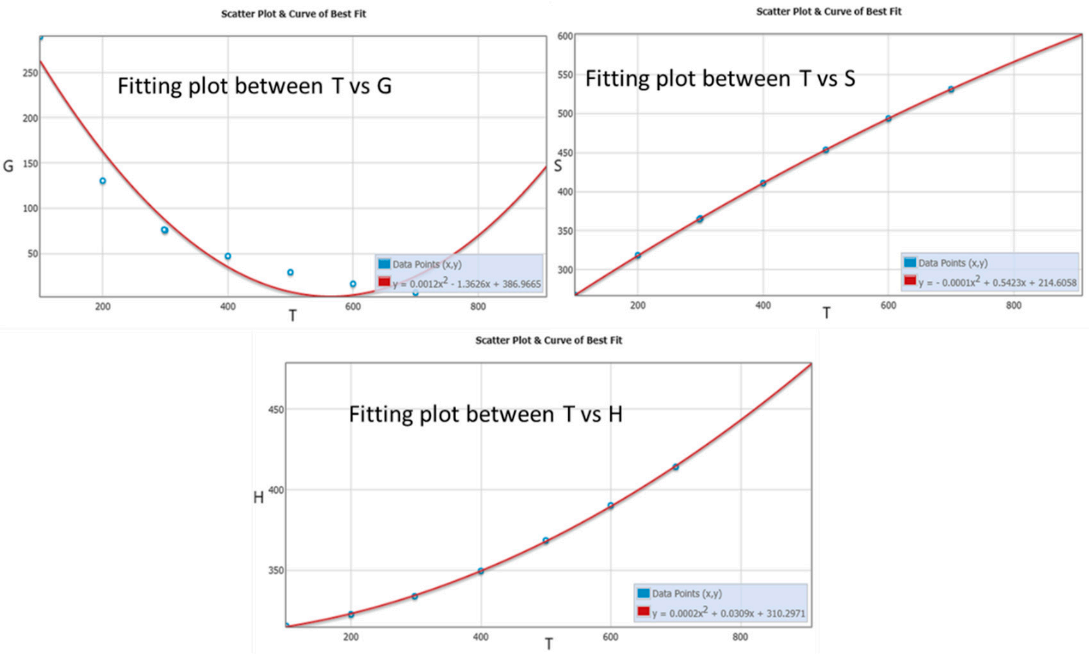

Figure S5. The plot showing relationships of temperature with Gibbs free energy, and entropy, enthalpy.

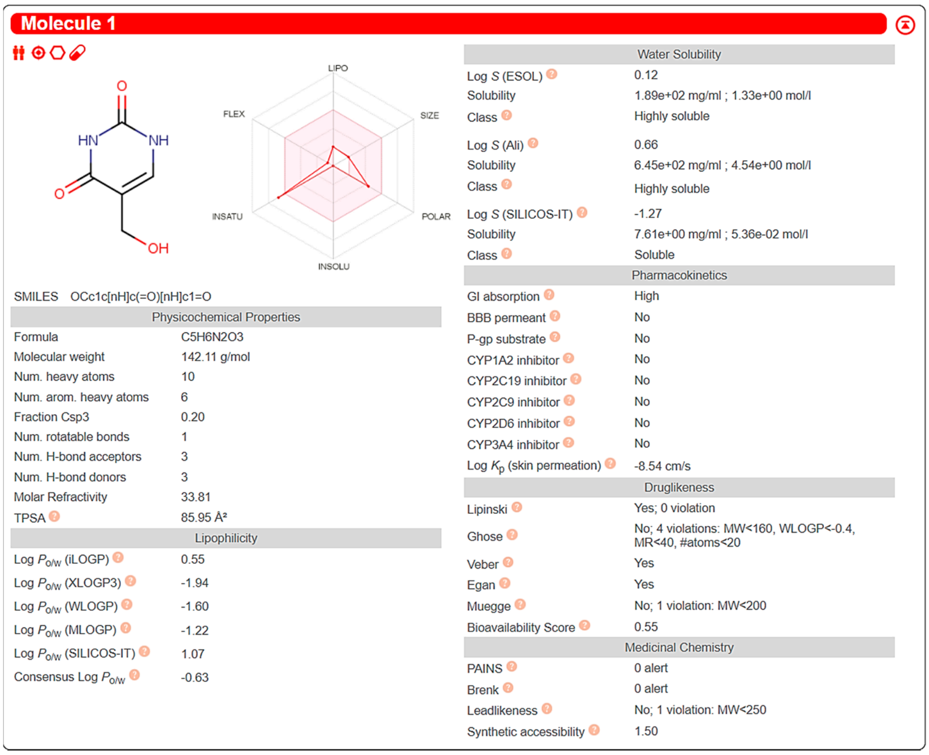

Figure S6. Predicted Lipinski's rule of five showing bioavailability radar plot of 5-HMU.

Table S1. Some optimized "Geometrical parameters" Bond Length (pm) and Bond Angle (º) of dimer **1**.

**"Bond length" (Å)**		**Bond angle (º)**	
Parameter	B3LYP/6-311++G(d,p)	Parameter	B3LYP/6-311++G(d,p)
O12-H18	1.85	N20-H18-O12	170.34
O19-H9	1.84	N6-H9-O17	170.28
N20-H18	1.03	H18-O12-C10	132.36
O20-C10	1.23	H9-O17-C19	132.42

Table S2. Some optimized "Geometrical parameters" Bond Length (pm) and Bond Angle (º) of dimer **2**.

**"Bond length" (Å)**		**Bond angle (º)**	
Parameter	B3LYP/6-311++G(d,p)	Parameter	B3LYP/6-311++G(d,p)
O5-H18	1.80	N20-H18-O5	174.05
O17-H2	1.80	N1-H2-O17	173.93
N20-H18	1.03	H18-O5-C3	126.13
O17-C19	1.23	H2-O17-C19	126.13

Table S3. Surface property information in Hirshfeld for 5-HMU.

Mode	Minimum interaction	Mean interaction	Maximum interaction
d_{norm}	-0.6245	0.303	0.9768
d_i	0.7259	1.5655	2.3342
d_e	0.7281	1.587	2.2269
Shape index	-0.9922	0.3242	0.9975
Curvedness	-4.043	-0.971	0.306
Fragment patches	0	6.5113	13

Table S4. Second-order perturbation theory of the Fock matrix NBO analysis of 5HMU.

**Donor**	**Type**	**ED/e**	**Acceptor**	**Type**	**ED/e**	**E(2) kcal/mol**	**E(j)-E(i) a.u.**	**F(i,j) a.u.**
C3 - C4	π	1.84451	C5 - O7	π*	0.30494	22.5	0.3	0.077
N2	LP (1)	1.65394	C1 - O8	π*	0.35772	53.48	0.29	0.111
	LP (1)		C3 - C4	π*	0.19836	38.69	0.31	0.101
N6	LP (1)	1.64894	C1 - O8	π*	0.35772	58	0.28	0.113
	LP (1)		C5 - O7	π*	0.30494	48.98	0.29	0.107
O7	LP (2)	1.86299	C4 - C5	π*	0.05986	17.17	0.72	0.101
	LP (2)		C5 - N6	π*	0.08754	28.29	0.65	0.123
O8	LP (2)	1.84177	C1 - N2	π*	0.08373	26.09	0.66	0.119
	LP (2)		C1 - N6	π*	0.08091	25.49	0.67	0.119

Table S5. Hybrid, polarization coefficient and atomic orbital contribution in selected natural bond orbitals of 5HMU.

Bond Orbital	Hybrid A (h_A)	Atomic Orbital (%)	Polarization Coefficient (C_A)	Hybrid B (h_B)	Atomic Orbital (%)	Polarization Coefficient (C_B)
σ C1 - N2	$sp^{2.16}$	s(31.61%)p(68.28%)d(0.11%)	0.6112	$sp^{1.90}$	s(34.43%)p(65.52%)d(0.05%)	0.7915
σ C1 - N6	$sp^{2.10}$	s(32.26%)p(67.62%)d(0.11%)	0.6147	$sp^{1.80}$	s(35.75%)p(64.20%)d(0.05%)	0.7888
σ C1 - O8	$sp^{1.78}$	s(35.94%)p(63.93%)d(0.14%)	0.6043	$sp^{1.54}$	s(39.29%)p(60.59%)d(0.12%)	0.7968
π C1 - O8	$sp^{1.00}$	s(0.00%)p(99.49%)d(0.51%)	0.5351	$sp^{1.00}$	s(0.00%)p(99.88%)d(0.12%)	0.8448
σ N2 - C3	$sp^{1.64}$	s(37.93%)p(62.03%)d(0.04%)	0.7891	$sp^{2.52}$	s(28.41%)p(71.48%)d(0.11%)	0.6143
σ N2 - C3	$sp^{1.64}$	s(37.93%)p(62.03%)d(0.04%)	0.7891	$sp^{2.52}$	s(28.41%)p(71.48%)d(0.11%)	0.6143
σ N2 - H11	$sp^{2.62}$	s(27.58%)p(72.38%)d(0.04%)	0.8441	$sp^{0.00}$	1s(99.93%)p(0.07%)	0.5362
σ C3 - C4	$sp^{1.45}$	s(40.72%)p(59.24%)d(0.04%)	0.7048	$sp^{1.74}$	s(36.41%)p(63.54%)d(0.05%)	0.7094
π C3 - C4	$sp^{1.00}$	s(0.00%)p(99.92%)d(0.08%)	0.671	$sp^{1.00}$	s(0.01%)p(99.93%)d(0.06%)	0.7415
σ C4 - C5	$sp^{2.24}$	s(30.82%)p(69.13%)d(0.06%)	0.7155	$sp^{1.63}$	s(38.07%)p(61.89%)d(0.04%)	0.6986
σ C4 - C9	$sp^{2.05}$	s(32.75%)p(67.22%)d(0.02%)	0.7231	$sp^{2.32}$	s(30.12%)p(69.83%)d(0.05%)	0.6907
σ C5 - N6	$sp^{2.50}$	s(28.51%)p(71.37%)d(0.12%)	0.6062	$sp^{1.71}$	s(36.84%)p(63.12%)d(0.04%)	0.7953
σ C5 - O7	$sp^{2.01}$	s(33.18%)p(66.67%)d(0.15%)	0.6001	$sp^{1.47}$	s(40.43%)p(59.45%)d(0.12%)	0.7999
π C5 - O7	$sp^{1.00}$	s(0.00%)p(99.55%)d(0.45%)	0.5497	$sp^{1.00}$	s(0.00%)p(99.88%)d(0.12%)	0.8353
σ N6 - H13	$sp^{2.66}$	s(27.33%)p(72.62%)d(0.04%)	0.8458	$sp^{0.00}$	s(99.93%)p(0.07%)	0.5335
σ C9 -	$sp^{3.54}$	s(21.99%)p(77.80%)d(0.	0.5787	$sp^{2.35}$	s(29.85%)p(70.07%)d(0.	0.8155

O10		21%)			08%)	
σ O10 - H16	$sp^{3.84}$	s(20.64%)p(79.27%)d(0.09%)	0.8556	$sp^{0.00}$	s(99.89%)p(0.11%)	0.5176
LP(2) N2	$sp^{1.00}$	s(0.00%)p(99.99%)d(0.01%)				
LP(2) N6	$sp^{1.00}$	s(0.00%)p(99.99%)d(0.01%)				
LP(2) O7	$sp^{0.68}$	s(59.62%)p(40.37%)d(0.02%)				
LP(2) O7	$sp^{99.99}$	s(0.03%)p(99.89%)d(0.08%)				
LP(2) O8	$sp^{0.64}$	s(60.83%)p(39.16%)d(0.01%)				
LP(2) O8	$sp^{1.00}$	s(0.00%)p(99.91%)d(0.09%)				
LP(2) O10	$sp^{1.02}$	s(49.45%)p(50.52%)d(0.03%)				
LP(2) O10	$sp^{99.99}$	s(0.07%)p(99.88%)d(0.05%)				

Table S6. Natural hybrid orbital directionality and bond bending (deviations from line of nuclear centres) of 5HMU.

Bond Orbital	Deviation angle (o)		Line of Centers	
	Hybrid A	Hybrid B	Polar (ϴ)	Azimuthal (ϕ)
σ C1 - N2	5.3	1.2	87.1	229.5
σ C1 - N6	5	2.4	91	116.9
π C1 - O8	89.7	89.7	91.7	352.7
π C3 - C4	90.2	90.4	91.4	115.7
σ C5 - N6	1.5	2.3	91.8	348.8
π C3 - C4	90.2	90.4	91.4	115.7
π C5 - O7	90	89.9	90.9	109.3
σ C9 - O10	3.7	1.5	99.5	238.9
π C1 - O8	89.7	89.7	91.7	352.7
π C3 - C4	90.2	90.4	91.4	115.7

Table S7. Natural Hybrid Orbital directionality and bond bending (deviations from line of nuclear centres) of 5HMU.

Bond Orbital	Deviation angle (o)		Line of centers	
	Hybrid A	Hybrid B	Polar (ϴ)	Azimuthal (ϕ)
σ C1 - N2	5.3	1.2	87.1	229.5
σ C1 - N6	5	2.4	91	116.9
π C1 - O8	89.7	89.7	91.7	352.7
π C3 - C4	90.2	90.4	91.4	115.7
σ C5 - N6	1.5	2.3	91.8	348.8
π C3 - C4	90.2	90.4	91.4	115.7
π C5 - O7	90	89.9	90.9	109.3
σ C9 - O10	3.7	1.5	99.5	238.9
π C1 - O8	89.7	89.7	91.7	352.7
π C3 - C4	90.2	90.4	91.4	115.7

Table S8. Mulliken charge distribution, Fukui function, and local softness corresponding to (0,1), (−1,2), and (1,2) charge and multiplicity of 5-HMU.

Atom	Mulliken Atomic Charges			Fukui Functions				Local Softness		
	N (0,1)	N-1 (+1,2)	N+1 (-1,2)	F_{r+}	f_{r-}	Δf	f_{r0}	$s_{r+} f_{r+}$	$s_{r-} f_{r-}$	$s_{r0} f_{r0}$
C1	0.335664	0.323833	0.692518	0.356854	0.011831	0.345023	0.184343	0.131679	0.004366	0.068022
N2	-0.35916	-0.25402	-0.26158	0.097572	-0.10514	0.202713	-0.00378	0.036004	-0.0388	-0.0014
C3	0.005795	0.002215	0.056256	0.050461	0.00358	0.046881	0.027021	0.01862	0.001321	0.009971
C4	0.191845	0.342	-0.42715	-0.619	-0.15016	-0.46884	-0.38458	-0.22841	-0.05541	-0.14191
C5	0.008182	0.028508	-0.66595	-0.67413	-0.02033	-0.6538	-0.34723	-0.24875	-0.0075	-0.12813
N6	-0.40137	-0.38094	-0.38901	0.012358	-0.02042	0.03278	-0.00403	0.00456	-0.00754	-0.00149
O7	-0.32107	-0.17743	-0.40432	-0.08325	-0.14364	0.060396	-0.11344	-0.03072	-0.053	-0.04186
O8	-0.34027	-0.16814	-0.42585	-0.08558	-0.17213	0.086544	-0.12886	-0.03158	-0.06351	-0.04755
C9	-0.41924	-0.42743	2.262548	2.681792	0.008186	2.673606	1.344989	0.989581	0.003021	0.496301
O10	-0.2967	-0.24564	-0.27716	0.019537	-0.05106	0.070598	-0.01576	0.007209	-0.01884	-0.00582

The Fukui equations are:
Nucleophilic attack $f^{+}(r) = q_r(N+1) - q_r(N)$
Electrophilic attack $f^{-}(r) = q_r(N) - q_r(N-1)$
Radical attack $f^{0}(r) = [q_r(N+1) - q_r(N-1)]/2$
where q_r is the charge on the atom at the rth atomic site. (N) is neutral, (N+1) is anionic and (N-1) is cationic chemical species. 0, +, - sign indicated the radical, nucleophilic, and electrophilic attacks. Dual descriptors [Δf(r)] can be calculated as [16]:
$\Delta f(r) = f^{+}(r) - f^{-}(r)$

Table S9. Comparison of calculated and experimental electronic properties of 5-HMU.

Experimental			TD-B3LYP/6-311++G(d,p)			
**Solvent**	**Λ_{cal} (nm)**	**Band gap (eV)**	**λ_{cal} (nm)**	**Band Gap (eV)**	**Oscillatory Strength**	**Assignments**
**Gas**			261.67	4.7382	0.0028	H-1 →L (90%)
			247.86	5.0021	0.1286	H →L (89%)
			223.62	5.5443	0.0071	H →L+1 (97%)
**DMSO**	275	4.51	252.24	4.9153	0.1641	H →L (83%)
			246.68	5.0262	0.0351	H-1 →L (80%)
			213.48	5.8077	0.0321	H →L+1 (83%)
**Methanol**			251.81	4.9236	0.1482	H →L (79%)
			246.69	5.026	0.0409	H-1 →L (77%)
			213.46	5.8083	0.0292	H →L+1 (83%)

Table S10. Calculated energy values of 5-HMU.

Parameter	Values
E_{Homo}(eV)	-7.12
E_{Lumo}(eV)	-1.68
Ionization potential	7.12
Electron affinity	1.68
Energy gap(eV)	5.44
Electronegativity	4.4
Chemical potential	-4.4
Chemical hardness	2.72
Chemical softness	0.37
Electrophilicity index	3.56

Table S11. Calculated centroid coordinates and distances between EDD and HDD centroids for allowed excited state transition of 5-HMU in DMSO and methanol.

				**Centroid coordinates**			
**Solvent**	**Excited State**	**λ_{max}**	***f***	**Maps**	**X(Å)**	**Y(Å)**	**Z(Å)**
**DMSO**	1	252.24	0.1641	EDD	-0.10	0.02	0.05
				HDD	-0.07	-0.04	0.03
				Distance between Centroid	0.03	0.07	0.02
**MeOH**	1	251.81	0.1482	EDD	-0.10	0.02	0.05
				HDD	-0.08	0.00	0.03
				Distance between Centroid	0.01	0.01	0.02

Table S12. Temperature dependence of thermodynamic properties of 5-HMU at B3LYP/6–311++G(d,p).

T(K)	$G^0_{p,m}$ x10 (J/ mol K)	S^0_m (J/ mol K)	H^0_m (kJ/ mol)
100	289.6849	267.3434	315.5878
200	130.3866	318.399	322.7901
298.15	76.37102	364.7393	333.9682
400	47.17192	410.7919	349.6786
500	29.18838	453.6322	368.6007
600	16.50816	493.7711	390.3228
700	6.897448	531.1615	414.2749

Table S13. ADME properties of 5-HMU.

Properties	5-HMU
HBD	3
HBA	3
MR	33.81
TPSA (Å$^{2)}$	85.95
GI Absorption	High
BBB Permeant	No
CYP1A2 inhibitor	No
Log Kp (cm/s)	−8.54
Lipinski violations	0 violations
Bioavailability Score	0.55
MLogP	−1.22

Table S14. Hydrogen bonds occupancy with their donor–acceptor sides.

Donor	Acceptor	Occupancy
HIS42-Side	LIG578-Side	20.08%
LIG578-Side	HIS69-Side	43.91%
LIG578-Side	ASN205-Sid	2.25%
LIG578-Side	MET215-Main	2.60%
HIS208-Side	LIG578-Side	0.05%
HIS204-Side	LIG578-Side	0.15%
LIG578-Side	VAL218-Main	0.30%
ASN205-Side	LIG578-Side	0.25%
LIG578-Side	HIS208-Side	0.65%
LIG578-Side	HIS60-Side	0.45%
LIG578-Side	ALA59-Main	0.10%
LIG578-Side	HIS42-Side	0.05%
LIG578-Side	HIS42-Side	0.05%

References

1. Petersson, G.A.; Al-Laham, M.A. A complete basis set model chemistry. II. Open-shell systems and the total energies of the first-row atoms. *J. Chem. Phys.* **1991**, *94*, 6081–6090, doi:10.1063/1.460447.
2. Petersson, G.A.; Bennett, A.; Tensfeldt, T.G.; Al-Laham, M.A.; Shirley, W.A.; Mantzaris, J. A complete basis set model chemistry. I. The total energies of closed-shell atoms and hydrides of the first-row elements. *J. Chem. Phys.* **1988**, *89*, 2193–2218, doi:10.1063/1.455064.
3. M. J. Frisch, G. W. Trucks, H. B. Schlegel, G. E. Scuseria, M. A. Robb, J. R. Cheeseman, G. Scalmani, V. Barone, B. Mennucci, G. A. Petersson, H. Nakatsuji, M. Caricato, X. Li, H. P. Hratchian, A. F. Izmaylov, J. Bloino, G. Zheng, J. L. Sonnenberg, M. Hada, M. Ehara, K. Toyota, R. Fukuda, J. Hasegawa, M. Ishida, T. Nakajima, Y. Honda, O. Kitao, H. Nakai, T. Vreven, J. A. Montgomery, Jr., J. E. Peralta, F. Ogliaro, M. Bearpark, J. J. Heyd, E. Brothers, K. N. Kudin, V. N. Staroverov, R. Kobayashi, J. Normand, K. Raghavachari, A. Rendell, J. C. Burant, S. S. Iyengar, J. Tomasi, M. Cossi, N. Rega, J. M. Millam, M. Klene, J. E. Knox, J. B. Cross, V. Bakken, C. Adamo, J. Jaramillo, R. Gomperts, R. E. Stratmann, O. Yazyev, A. J. Austin, R. Cammi, C. Pomelli, J. W. Ochterski, R. L. Martin, K. Morokuma, V. G. Zakrzewski, G. A. Voth, P. Salvador, J. J. Dannenberg, S. Dapprich, A. D. Daniels, O. Farkas, J. B. Foresman, J. V. Ortiz, J. Cioslowski, and D. J. Fox, Gaussian, Inc., Wallingford CT, 2009, Gaussian 09, Revision A.02.
4. Neese, F. The ORCA program system. *WIREs Comput. Mol. Sci.* **2012**, *2*, 73–78, doi:10.1002/wcms.81.
5. Jamróz, M.H. Vibrational Energy Distribution Analysis (VEDA): Scopes and limitations. *Spectrochim. Acta Part A Mol. Biomol. Spectrosc.* **2013**, *114*, 220–230, doi:10.1016/j.saa.2013.05.096.
6. Lu, T.; Chen, F. Multiwfn: A multifunctional wavefunction analyzer. *J. Comput. Chem.* **2012**, *33*, 580–592, doi:10.1002/JCC.22885.
7. Safin, D.A.; Robeyns, K.; Garcia, Y. 1,2,4-Triazole-based molecular switches: crystal structures, Hirshfeld surface analysis and optical properties. *CrystEngComm* **2016**, *18*, 7284–7296, doi:10.1039/C6CE00749J.
8. Spackman, M.A.; Jayatilaka, D. Hirshfeld surface analysis. *CrystEngComm* **2009**, *11*, 19–32, doi:10.1039/B818330A.
9. Wang, W.; Ling, Y.; Yang, L.-J.; Liu, Q.-L.; Luo, Y.-H.; Sun, B.-W. Crystals of 4-(2-benzimidazole)-1,2,4-triazole and its hydrate: preparations, crystal structure and Hirshfeld surfaces analysis. *Res. Chem. Intermed.* **2016**, *42*, 3157–3168, doi:10.1007/s11164-015-2203-2.
10. Shyamapada, S.; Christoph, M.; Mitra, S. Synthesis, Crystal structure, and Hirshfeld Surface Analysis of a New Mixed Ligand Copper(II) Complex. *Acta Chim. Slov.* **2016**, *63*, 129–137, doi:10.17344/acsi.2015.2024.
11. Chohan, Z.H.; Youssoufi, M.H.; Jarrahpour, A.; Ben Hadda, T. Identification of antibacterial and antifungal pharmacophore sites for potent bacteria and fungi inhibition: Indolenyl sulfonamide derivatives. *Eur. J. Med. Chem.* **2010**, *45*, 1189–1199, doi:10.1016/j.ejmech.2009.11.029.
12. Ben M’leh, C.; Brandán, S.A.; Issaoui, N.; Roisnel, T.; Marouani, H. Synthesis, molecular structure, vibrational and theoretical studies of a new non-centrosymmetric organic sulphate with promising NLO properties. *J. Mol. Struct.* **2018**, *1171*, 771–785, doi:10.1016/j.molstruc.2018.06.041.
13. Trott, O.; Olson, A.J. AutoDock Vina: Improving the speed and accuracy of docking with a new scoring function, efficient optimization, and multithreading. *J. Comput. Chem.* **2009**, doi:10.1002/jcc.21334.
14. Pettersen, E.F.; Goddard, T.D.; Huang, C.C.; Couch, G.S.; Greenblatt, D.M.; Meng, E.C.; Ferrin, T.E. UCSF Chimera, A visualization system for exploratory research and analysis. *J. Comput. Chem.* **2004**, *25*, 1605–1612, doi:10.1002/jcc.20084.
15. Daina, A.; Michielin, O.; Zoete, V. SwissADME: A free web tool to evaluate pharmacokinetics, drug-likeness and medicinal chemistry friendliness of small molecules. *Sci. Rep.* **2017**, *7*, 42717, doi:10.1038/srep42717.
16. Yang, W.; Mortier, W.J. The use of global and local molecular parameters for the analysis of the gas-phase basicity of amines. *J. Am. Chem. Soc.* **1986**, *108*, 5708–5711, doi:10.1021/ja00279a008.
